# Supplementary figures and images for: The endogenous cellular protease inhibitor SPINT2 controls SARS-CoV-2 viral infection and is associated to disease severity
Source: PLoS Pathog. 2021 Jun 28;17(6):e1009687. doi: 10.1371/journal.ppat.1009687 (PMC8270430; doi:10.1371/journal.ppat.1009687)

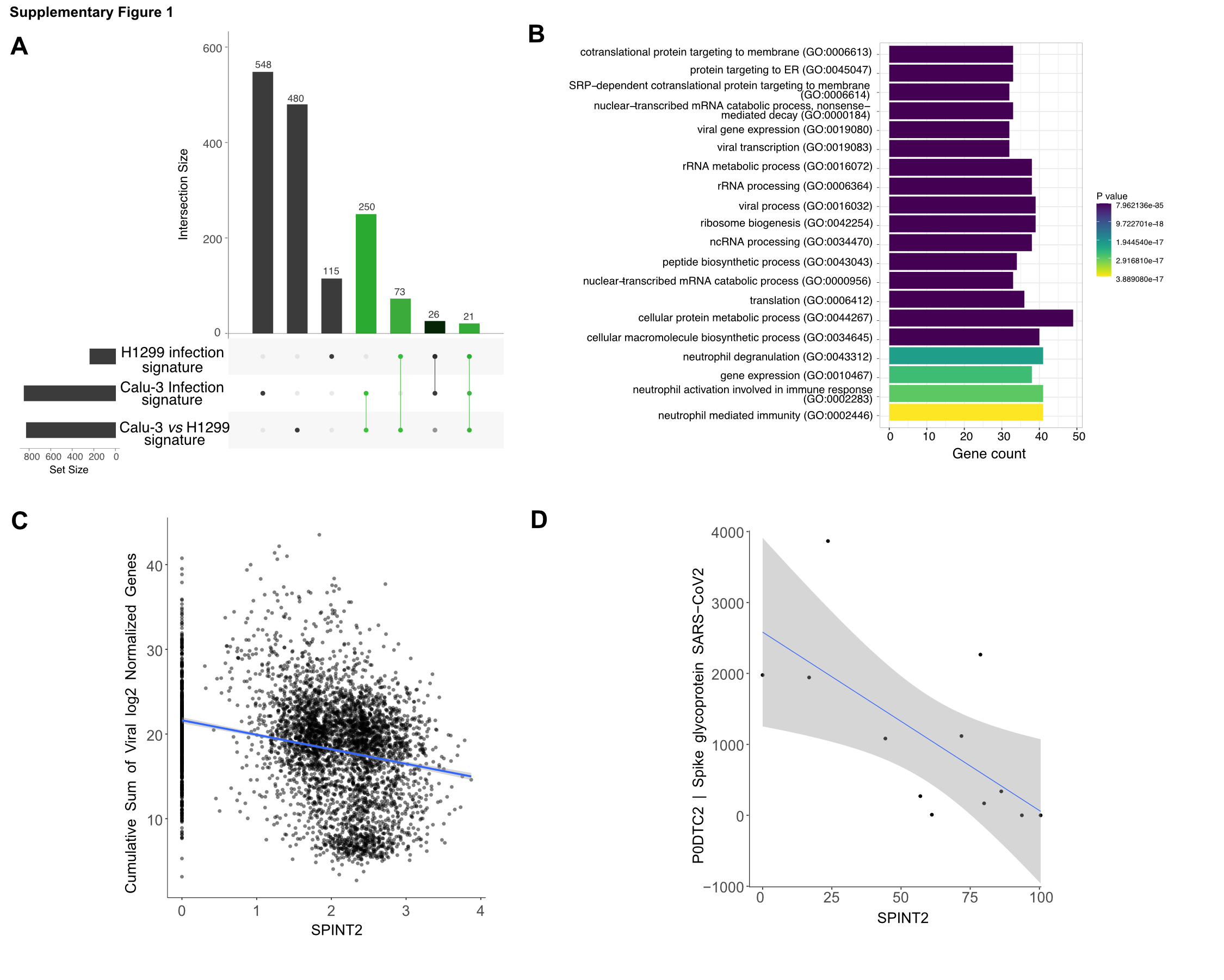

Supplement: S1 Fig — A. Upset plot showing the intersections of Calu-3 vs H1299 DEGs with Calu-3 and H1299 viral-induced genes signatures (see Methods). Removed genes from the permissivity signature are shown in green. B. Gene set enrichment of filtered out genes (green intersections in A). C. Correlation of SPINT2 to the cumulative sum of normalized expression values of SARS-CoV-2 genes in Calu-3 cells. D. Correlation of SPINT2 to S viral protein translation rates in Caco-2 cells. (TIFF) [file ppat.1009687.s001.tiff]

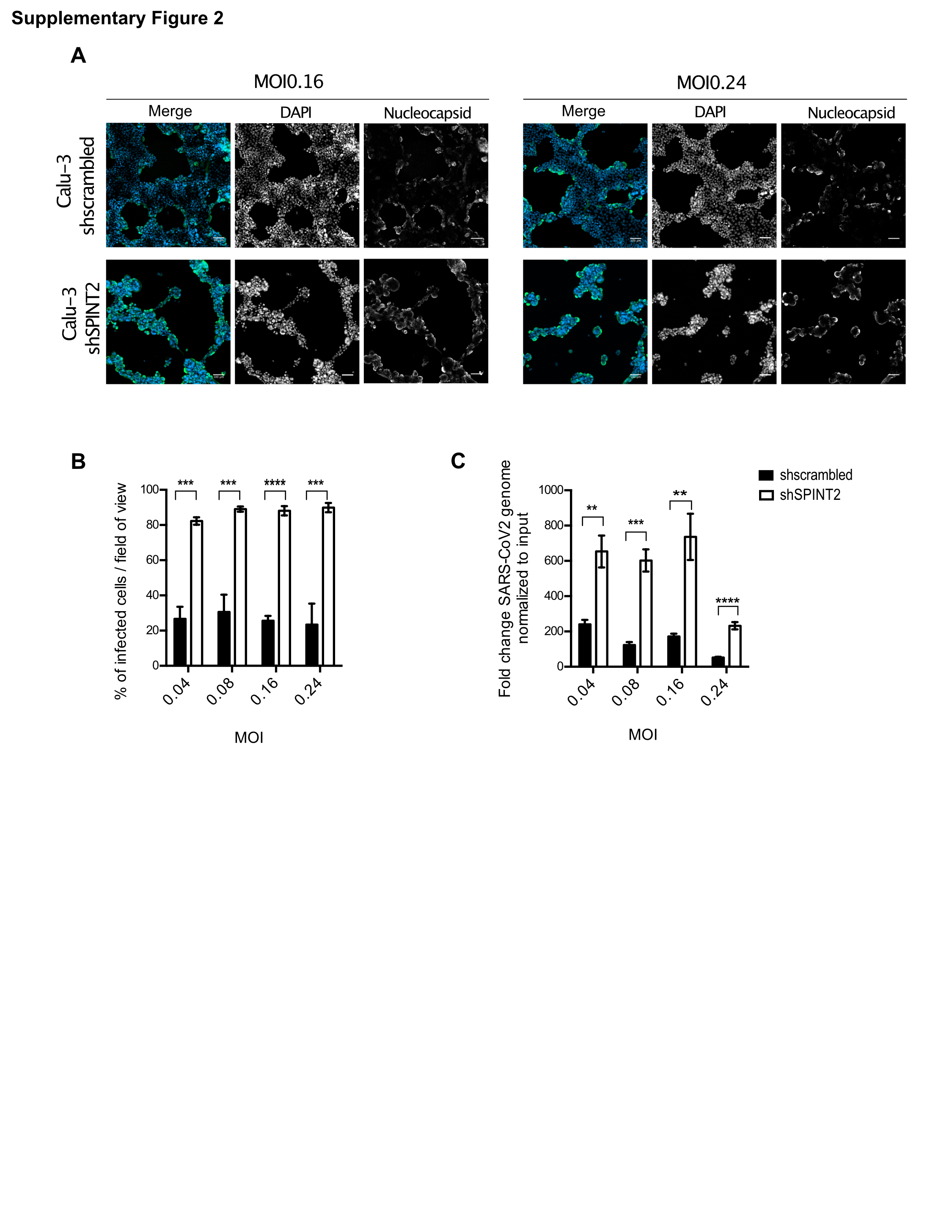

Supplement: S2 Fig — A. Representative immunofluorescence images of Calu-3 cells treated with scrambled control shRNA (sh-scrambled) and shRNA targeting SPINT2 (shSPINT2) infected with SARS-CoV-2 at indicated MOI (as determined in Vero cells). Infection was detected by indirect immunofluorescence against the nucleocapsid N and Nuclei were stained with DAPI. MOI 0.16 and MOI 0.24 are shown. B. Quantification of the percentage of SARS-CoV-2 infected cells at the indicated MOI for cells treated with scrambled control shRNA (sh-scrambled) and shRNA targeting SPINT2 (shSPINT2). C. Quantification of SARS-CoV-2 replication as in D. RNA was harvested at 24 hpi, and q-RT-PCR was used to evaluate the copy number of the SARS-CoV-2 genome. Data are normalized to inoculum used for infection for each MOI. Error bars indicate standard deviation. n = 3 biological replicates. P<0.05 *, P<0.01 **, P<0.001 ***, P<0.0001 ****. Analysis was done by a two-tailed unpaired t-test with Welch’s correlation for the respective MOI. (TIFF) [file ppat.1009687.s002.tiff]

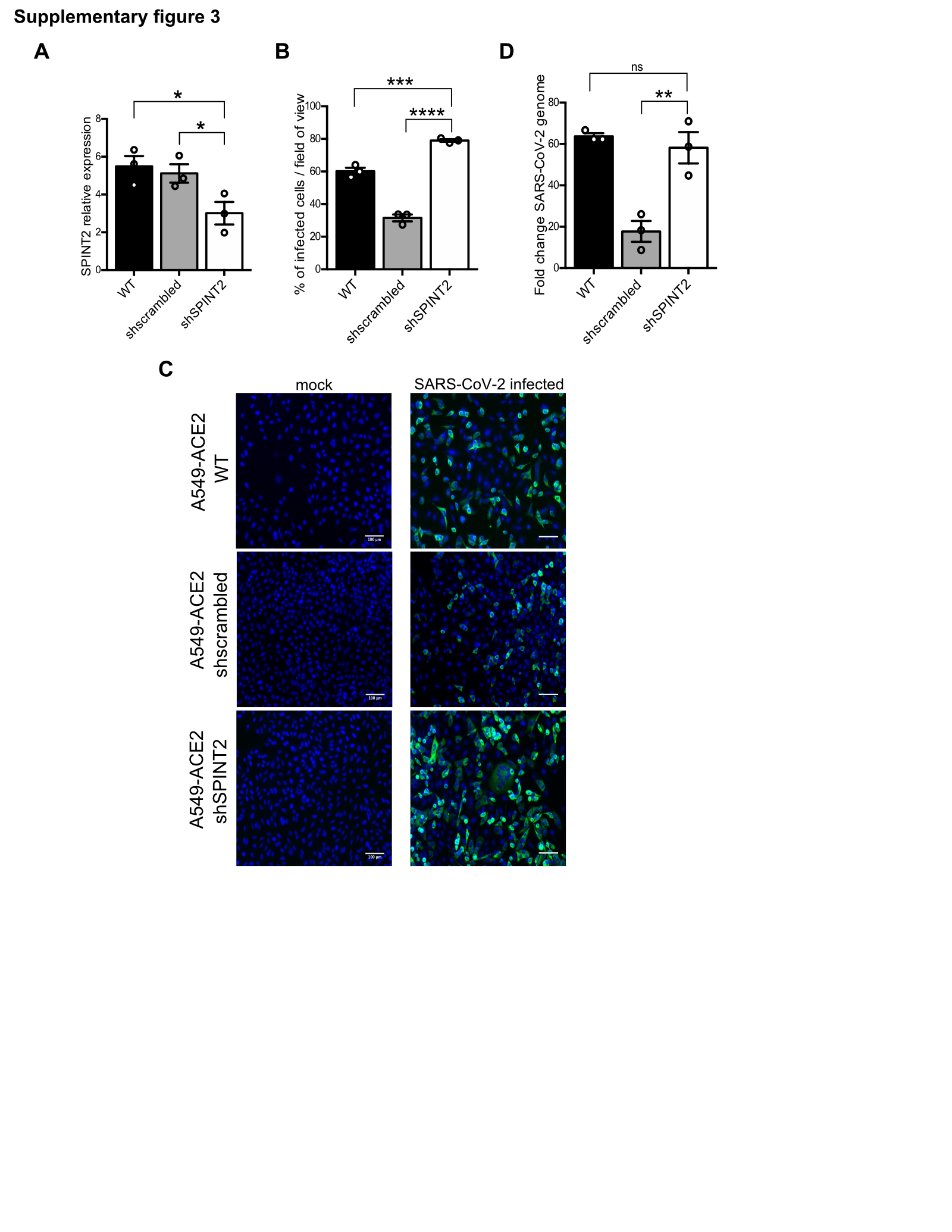

Supplement: S3 Fig — A. Relative expression of SPINT2 normalized to the housekeeping gene TBP in wild type, sh-scrambled and SPINT2 KD A549-ACE2 cells. B. Percentage of infected cells in A549-ACE2 wild type (WT) cells and cells treated with scrambled control shRNA (sh-scrambled) and shRNA targeting SPINT2 (shSPINT2) for mock-infected and SARS-CoV-2 infected conditions. Infection was detected by indirect immunofluorescence against the nucleocapsid N and Nuclei were stained with DAPI. C. Representative immunofluorescence images of the cells shown in B. D. Quantification of SARS-CoV-2 replication. RNA was harvested at 24 hpi, and q-RT-PCR was used to evaluate the copy number of the SARS-CoV-2 genome. Data are normalized to inoculum used for infection. Error bars indicate standard deviation. n = 3 biological replicates. P<0.05 *, P<0.01 **, P<0.001 ***, P<0.0001 ****, Analysis was done by a two-tailed unpaired t-test with Welch’s correlation using SPINT2 KD A549 cells as reference. (TIFF) [file ppat.1009687.s003.tiff]

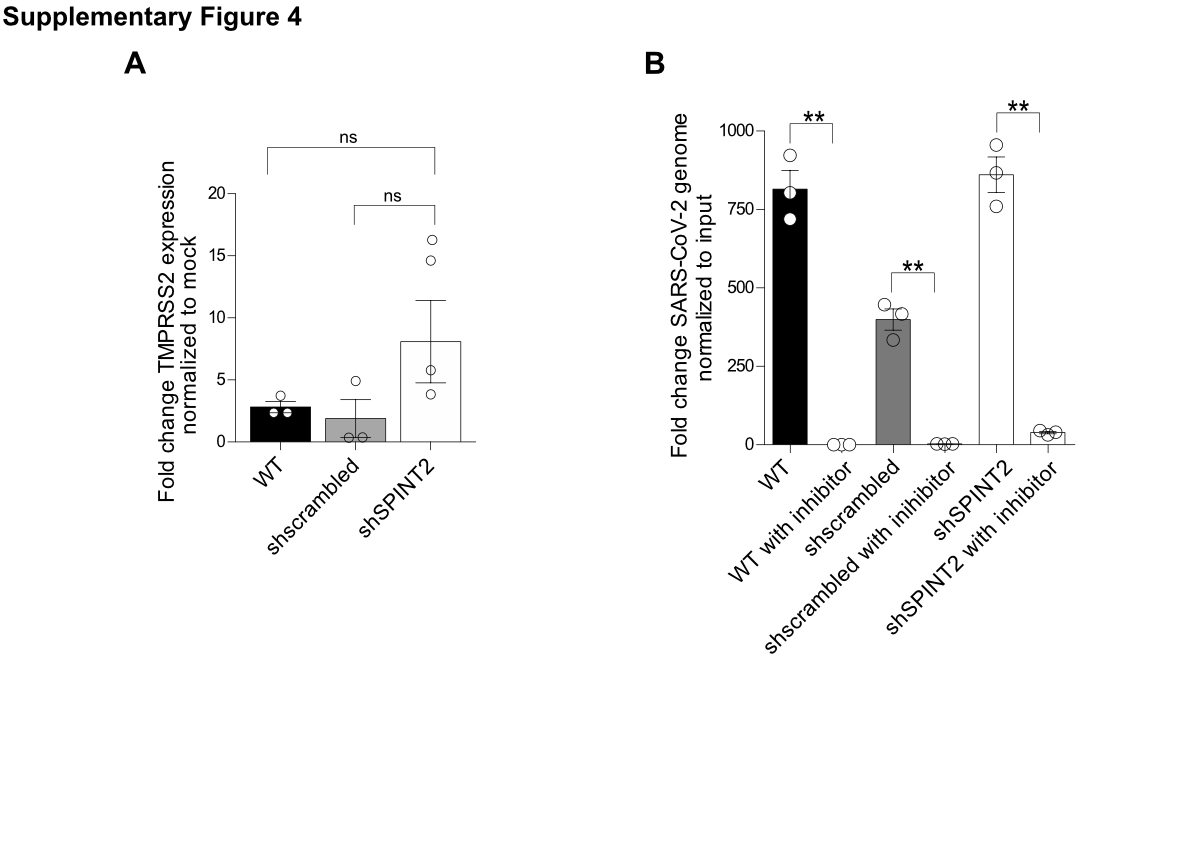

Supplement: S4 Fig — A. Quantification of TMPRSS2 expression upon SPINT2 silencing. q-RT-PCR was used to evaluate the expression level of TMPRSS2 in WT, sh-scrambled and SPINT2 KD in A549 cells transduced with ACE2. Relative expression level of TMPRSS2 in SARS-CoV-2 infected cells normalized to that of the mock infected cells. B. Quantification of SARS-CoV-2 replication upon SPINT2 KD and TMPRSS2 inhibition conditions in Calu-3 cells. RNA was harvested at 24 hpi and q-RT-PCR was used to evaluate the copy number of the SARS-CoV-2 genome. Data are normalized to inoculum used for infection. Error bars indicate standard deviation. n = 3 biological replicates. P<0.05 *, P<0.01 **, P<0.001 ***, P<0.0001 ****. Analysis was done by a two-tailed unpaired t-test with Welch’s correlation for the respective cell lines. (TIFF) [file ppat.1009687.s004.tiff]

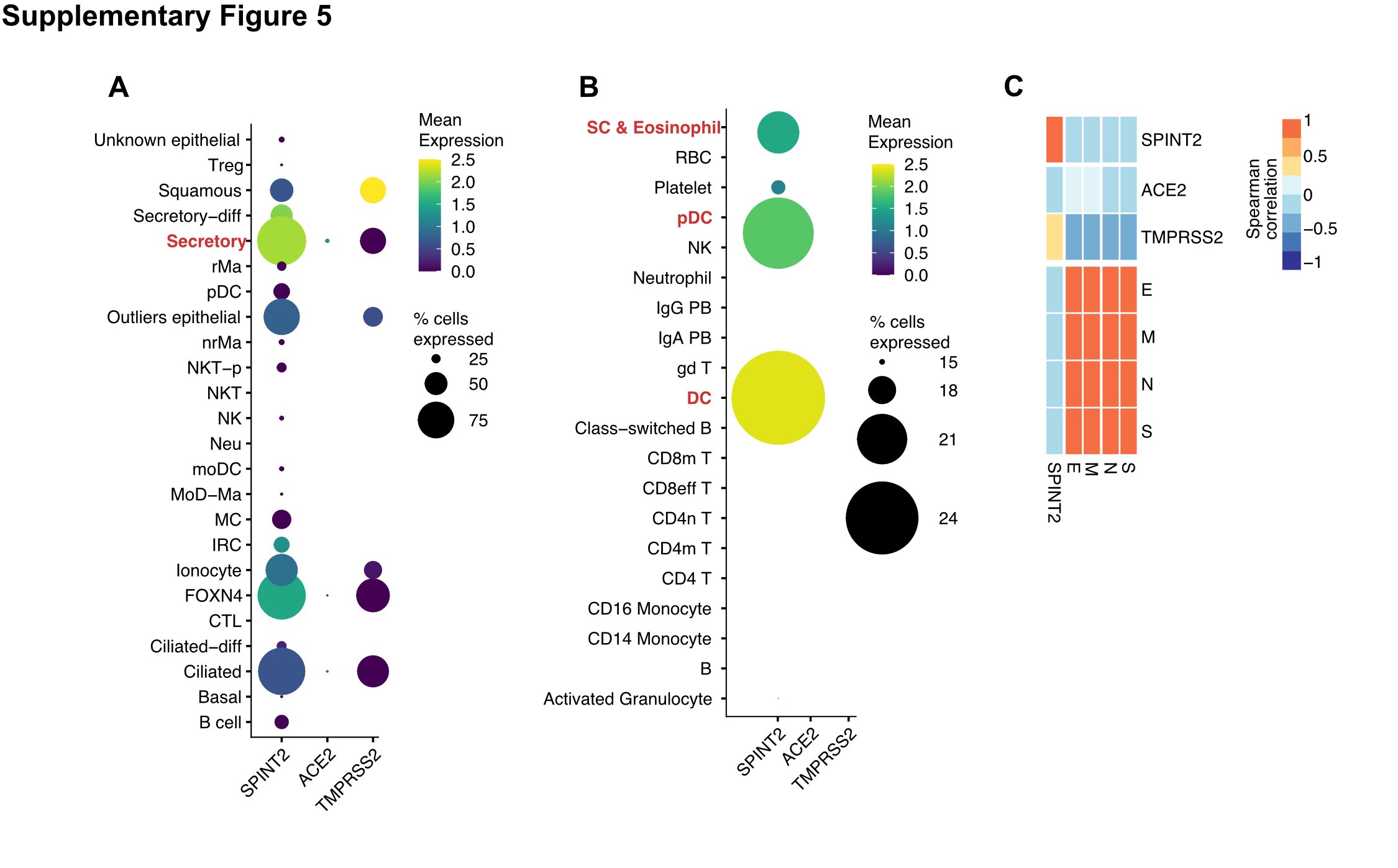

Supplement: S5 Fig — ACE2, SPINT2 and TMPRSS2 expression in Chua RL et al, 2020 (A) and Blish C et al, 2020 (B) scRNA-seq datasets. SPINT2 expressing cell types shown in the main text are highlighted in red for both datasets. C. Correlation of ACE2, SPINT2 and TMPRSS2 to viral proteins using bulk RNA-seq from COVID-19 deceased patients in Desai N et al, 2020. (TIFF) [file ppat.1009687.s005.tiff]

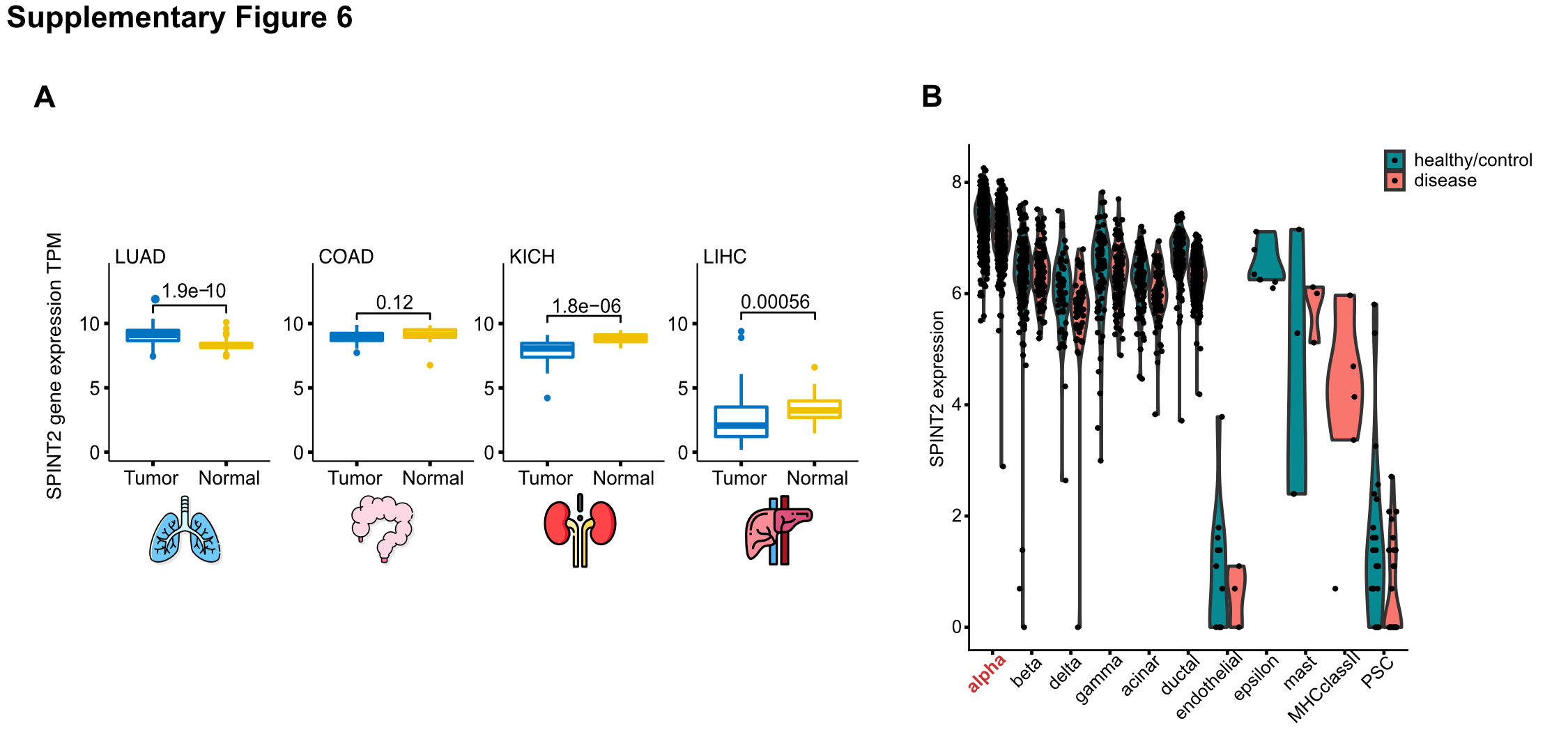

Supplement: S6 Fig — A. Normalized gene expression (TPM) of SPINT2 in different cancers. P-values from Wilcoxon Test. B. SPINT2 gene expression in pancreatic cells from diabetic patients. Hepatocytes and pancreatic alpha cells clusters highlighted in red represent cells for which gene expression profiles are shown in Fig 5. (TIFF) [file ppat.1009687.s006.tiff]
